# Deficits in Water Maze Performance and Oxidative Stress in the Hippocampus and Striatum Induced by Extremely Low Frequency Magnetic Field Exposure

**DOI:** 10.1371/journal.pone.0032196

**Published:** 2012-05-03

**Authors:** Yonghua Cui, Zhiqiang Ge, Joshua Dominic Rizak, Chao Zhai, Zhu Zhou, Songjie Gong, Yi Che

**Affiliations:** 1 Medical College of Soochow University, Suzhou, People’s Republic of China; 2 Laboratory of Primate Neuroscience Research, Key Laboratory of Animal Models, Kunming Institute of Zoology, Chinese Academy of Science, Kunming, People’s Republic of China; Mental Health Research Institute and the University of Melbourne, Australia

## Abstract

The exposures to extremely low frequency magnetic field (ELF-MF) in our environment have dramatically increased. Epidemiological studies suggest that there is a possible association between ELF-MF exposure and increased risks of cardiovascular disease, cancers and neurodegenerative disorders. Animal studies show that ELF-MF exposure may interfere with the activity of brain cells, generate behavioral and cognitive disturbances, and produce deficits in attention, perception and spatial learning. Although, many research efforts have been focused on the interaction between ELF-MF exposure and the central nervous system, the mechanism of interaction is still unknown. In this study, we examined the effects of ELF-MF exposure on learning in mice using two water maze tasks and on some parameters indicative of oxidative stress in the hippocampus and striatum. We found that ELF-MF exposure (1 mT, 50 Hz) induced serious oxidative stress in the hippocampus and striatum and impaired hippocampal-dependent spatial learning and striatum-dependent habit learning. This study provides evidence for the association between the impairment of learning and the oxidative stress in hippocampus and striatum induced by ELF-MF exposure.

## Introduction

The exposures to extremely low frequency magnetic field (ELF-MF) in our environment have dramatically increased, which include both occupational exposure and general exposure to sources, such as power lines, household electrical wiring and medical devices [1]. Epidemiological studies suggest that there is a possible association between ELF-MF exposure and increased risks of cardiovascular disease, cancers and neurodegenerative disorders [2]–[6]. Additionally, animal studies have shown that ELF-MF exposure (50 Hz, 1 mT) generated behavioral and cognitive disturbances, and produced deficits in attention, perception and spatial learning in rats [7]. Our research group has also demonstrated that ELF-MF exposure (50 Hz, 1 mT) induced significant impairments in detour learning and one-trial passive avoidance learning in chicks [8], [9].

Although, many research efforts have been focused on the interaction between ELF-MF exposure and the central nervous system, the mechanism of interaction is still unknown [10], [11]. Some reports suggested that ELF-MF exposure may affect biological systems by increasing the life span and concentration of reactive oxygen species (ROS) and free radicals [12], [13], [14]. Lai and Singh reported that ELF-MF exposure (60 Hz, 0.01 mT) caused DNA damage in rat brain cells with the involvement of oxygen free radical processes [15], Ravera *et al.* reported that ELF-MF exposure (75 Hz, 0.74 mT) decreased the activity of different membrane-anchored enzymes [16], Amara *et al*. reported that subchronic static magnetic fields exposure (128 mT) induced oxidative stress in the rat hippocampus and frontal cortex [17], and Jelenkovic *et al*. reported that ELF-MF exposure (50 Hz, 0.5 mT) was harmful to the brain, especially to the basal forebrain and frontal cortex due to development of lipid peroxidation [18].

It is well known that the increases of free radicals lead to oxidative damage in major cell macromolecules such as proteins, lipids, and nucleic acids. The brain tissues, especially the hippocampal and striatum neurons, are considered particularly vulnerable to oxidative damage because of their high lipid content, enrichment in mitochondria, comparatively high oxygen utilization, and modest antioxidant defences [19].

Previous studies have emphasized the effects of ELF-MF exposure on learning behavior and on the antioxidant status of various tissues and organs. However, no reports have been found in the literature to provide direct evidence that the impairment of learning induced by ELF-MF exposure involves oxidative stress. Therefore, it seemed reasonable to verify the hypothesis that ELF-MF exposure induces oxidative stress in some brain areas, damages the brain structure and function, and then impairs learning ability. The present investigation was designed to evaluate the effects of chronic ELF-MF exposure on habit learning which is dependent on striatum [20], [21], and on spatial learning which is dependent on hippocampus [20], [22], as well as evaluate some parameters indicative of oxidative stress in these two structures. We believe that this study may provide more evidence for the hypothesis that the impairment of learning induced by ELF-MF exposure involves oxidative stress.

## Materials and Methods

### Ethics Statement

All experiments were conducted during the light phase, and in accordance with procedures approved by Animal Experimental Committee, Soochow University, and with the National Institutes of Health Guidelines for the Care and Use of Laboratory Animals (NIH Guidelines).

### Subjects

Seventy-two adult male C57BL/6 mice weighing 19∼21 g at the beginning of the experiment were housed in pairs in plexiglas cages with food and water *ad libitum* throughout the experimental period. The mice were maintained in a climate-controlled colony room at 24°Con a 12/12 h reverse light/dark cycle. All mice were allowed 1 week adaptation to the housing rooms prior to the initiation of treatment. After adapting, mice received ELF-MF exposure 4 hours/day for 12 consecutive weeks. Mice were randomly assigned to three groups (n = 24 animals/group). Group I received sham ELF-MF exposure 4 hours/day. Sham-exposed mice were placed in a similar, but non-energized apparatus. Group II received 50 Hz, 1 mT ELF-MF exposure 4 hours/day. Group III received 50 Hz, 0.1 mT ELF-MF exposure 4 hours/day.

After the exposure period, all mice were evaluated in an open-field test and with a water-maze learning and memory task. Following these evaluations, the animals were slightly anesthetized and sacrificed by decapitation. Their brains were rapidly removed, and the hippocampus and striatum were carefully dissected out according to a mouse brain atlas with a previously described method [23].

### Magnetic Field Exposure System

As previously described [9], the electromagnetic field was generated by a single coil of four layers, each having 250 turns. Each layer was wrapped horizontally above the previous layer around a 70 cm×40 cm×43 cm plastic frame. The coil was connected to a waveform generator for modulating the frequency and intensity of the electromagnetic field. By varying the input current to the coil, the flux density of electromagnetic fields in the exposure area can be adjusted from ambient levels to the maximum coil-designed electromagnetic field strength of 14 mT. The exposure area (60 cm×30 cm×43 cm) was inside the coil. During exposure, mice were placed in a plastic box (50 cm×25 cm, with 25 cm high walls) which was mechanically isolated from the magnet and rested on a freestanding wood. The mice were free to move about the box *ad libitum*. The variation of the electromagnetic fields in the plastic box was ±4.5% of the mean and was determined by measurement with a Gaussmeter.

### Open Field Test

In order to determine whether ELF-MF exposure produced ataxia or motor impairments in the mice, locomotor activity was monitored in an open field enclosed in a black Plexiglas square box (40×40×30 cm). The open field tests were conducted after the 12 weeks of ELF-MF exposure. On the test day, mice were placed in the center of the black Plexiglas square box (40×40×30 cm) for a 5 min. Mice activity was recorded with a video camera that was connected to a computer.

### Water Maze Test

The mice were submitted to two versions of the water maze task after the open field test. The water maze consisted of a round tank, 110 cm in diameter and 30 cm deep, filled with water. The water temperature was maintained at 25°C. Several visual cues were placed on the walls of the laboratory. The latency to reach the escape platform was recorded with a video camera that was connected to a computer.

Thirty-six mice (n = 12/group) were submitted to a spatial reference memory version of the water maze. This consisted of 4 training days, four consecutive trials per day, during which the animals were randomly left in the tank facing the wall, and allowed to swim freely to a transparent escape platform (8 cm in diameter) submersed 2 cm under the water surface, placed in the center of one of the four imaginary quadrants of the tank. The escape platform position was maintained constant throughout all 4 training days. After the animal escaped to the platform it was allowed to remain on it for 30 s and was then removed from the tank for 30 s before being placed in the next random initial position. If the animal did not find the platform during a period of 60 s it was gently guided to it [20].

The other thirty-six mice (n = 12/group) were submitted to a cue version of the water maze similar to the previous experimental procedure, except that the position of the escape platform was cued by a yellow ping-pong ball attached to the top of the platform and floating above the water. Additionally, the position of the platform was always changed in this version of the water maze for each trial of the day. This memory task was a model of habit learning [20].

### Determination of Oxidative Stress

Mouse hippocampus and striatum were homogenized in 10∶1 (vol/wt) ice-cold PBS. A quantity of the homogenate was used to determine the activities of catalase (CAT), glutathione peroxidase (GSH-PX), total antioxidant capability (T-AOC) and the concentration of malondialdehyde (MDA) in the hippocampus and striatum samples according to the manufacturer’s protocol (Nanjing Jiancheng Bioengineering Institute, Nanjing, China). These methods are described briefly below.

CAT is responsible for the detoxification of H_2_O_2_, a precursor for intracellular free radicals [24]. The activity of CAT in the samples was measured by the decrease in the H_2_O_2_ concentration. The H_2_O_2_ decomposition reaction catalysed by catalase was stopped by adding ammonium molybdate. The remaining H_2_O_2_ combined with ammonium molybdate to form a yellow compound, which absorbed maximally at 405 nm. One unit of catalase activity was defined as 1 mmol of decomposed H_2_O_2_ in one milligram of tissue for one minute and expressed as units per mg protein.

The T-AOC is a useful index for the capacity of tissue samples to modulate the damage associated with enhanced production of free radicals [25]. A spectrometric method was applied to evaluate the T-AOC. In the reaction mixture, ferric ions were reduced by antioxidant reducing agents and a blue complex Fe^2+^–TPTZ (2,4,6-tri(2-pyridyl)-s-triazine) was produced. One unit of T-AOC was equal to 0.01 increases in absorbance of the reaction mixture at 520 nm per milligram protein per minute under 37°C incubation. The T-AOC activities were expressed as units per mg protein.

GSH-PX is responsible for breaking down peroxides [24]. The activity of GSH-PX was measured by measuring the rate of glutathione oxidation by hydrogen peroxide (2GSH+H_2_O_2_→GSSG+2H_2_O) as catalyzed by GSH-PX present in the sample. The activity was measured with the addition of glutathione reductase and NADPH. Glutathione reductase converts oxidized glutathione (GSSG) to the reduced form, while oxidizing NADPH to NADP. The rate of GSSG formation was subsequently measured by following the decrease in absorbance of the reaction mixture at 340 nm as NADPH was converted to NADP. A GSH-PX unit was defined as the enzyme activity required to convert 1 nmol of NADPH to NADP per mg tissue protein. The GSH-PX activity was expressed as units per mg protein.

MDA is one of the most frequently used indicators of lipid peroxidation [26]. The thiobarbituric acid reaction (TBAR) method was used to determine the MDA. The method was used to obtain a spectrophotometric measurement of the color produced during the reaction of TBA with MDA at 535 nm. MDA content was expressed as nmol/mg protein.

Protein concentrations were determined according to the Lowry method [27].

### Statistical Analysis

Statistical analysis was performed using SPSS software. All results were expressed as mean ± standard error of the mean. Differences between groups in body weight, oxidative stress levels and the distance in open field were analyzed by one-way repeated measures analysis of variance (ANOVA) followed by a post hoc LSD test. Escape latencies in water maze were analyzed by two-way repeated measures ANOVA using treatment as the between-subjects factor and session day as the repeated measure. Differences were considered to be statistically significant when p<0.05.

## Results

### Body Weight and Locomotion

During the treatment, animals were all in the growth state. There was no significant difference in weight gain across the three groups [F(2,33) = 0.271, p = 0.764]. The effect of ELF-MF exposure on general locomotor activity of the mice was examined in an open field test and the ELF-MF exposure did not cause significant differences in horizontal locomotion [F(2,33) = 0.767, p = 0.516]. This finding suggests that magnetic field exposure did not produce ataxia or motor impairments in the mice.

### ELF-MF Exposure Induced Oxidative Stress in the Hippocampus and Striatum

**Figure 1:**
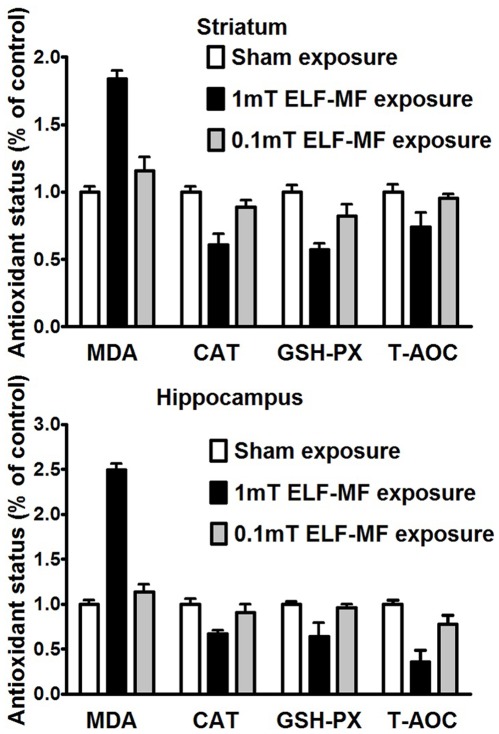
The Antioxidatant Status in Mice Hippocampus and Striatum. After extremely low frequency magnetic field (ELF-MF) exposure for consecutive 12 weeks, the antioxidatant status in mice hippocampus and striatum was impaired by ELF-MF exposure (1 mT) but not by ELF-MF exposure (0.1 mT). Values represent means±SEM. N = 12. * indicates p<0.05.

The levels of CAT, GSH-PX, T-AOC and MDA in mice hippocampus and striatum were determined in this study. ELF-MF exposure (0.1 mT) for 12 weeks did not result in the development of oxidative stress in mice hippocampus or striatum. However, ELF-MF exposure for 12 weeks (1 mT) resulted in the development of oxidative stress in mice hippocampus and striatum. The levels of MDA in mice hippocampus and striatum, parameters of oxidative stress, were significantly increased in the 1 mT ELF-MF exposure group compared with the other two groups [Hippocampus: F(2,33) = 40.62, p<0.001; striatum F(2,33) = 23.148, p<0.001]. The levels of CAT, GSH-PX, and T-AOC in hippocampus [CAT: F(2,33) = 7.765, p = 0.002; GSH-PX: F(2,33) = 9.151, p = 0.001; T-AOC: F(2,33) = 22.35, p<0.001] and striatum [CAT: F(2,33) = 16.31, p<0.001; GSH-PX: F(2,33) = 11.89, p<0.001; T-AOC: F(2,33) = 4.37, p = 0.021] were significantly declined in 1 mT ELF-MF exposure group compared with the other two groups (Fig 1, Table 1).

**Table 1:** The activities of CAT, GSH-PX, T-AOC and the concentration of MDA.

Group	CAT(U/mg protein)	GSH-PX(U/mg protein)	T-AOC(U/mg protein)	MDA(nmol/mg protein)
Hippocampus control	21.21±1.26	31.22±0.93	1.65±0.08	3.83±0.19
Hippocampus 1 mT	14.28±0.84*	20.15±4.65*	0.59±0.22*	9.57±0.26*
Hippocampus 0.1 mT	19.32±1.89	30.07±1.24	1.29±0.16	4.37±0.32
Striatum control	23.72±0.94	39.81±1.99	1.82±0.10	5.10±0.21
Striatum 1 mT	14.45±1.89*	22.68±1.99*	1.35±0.19*	9.38±0.31*
Striatum 0.1 mT	21.09±1.18	32.63±3.58	1.74±0.05	5.91±0.51

* p<0.05.

### ELF-MF Exposure Induced Learning Deficit in the Water Maze

**Figure 2:**
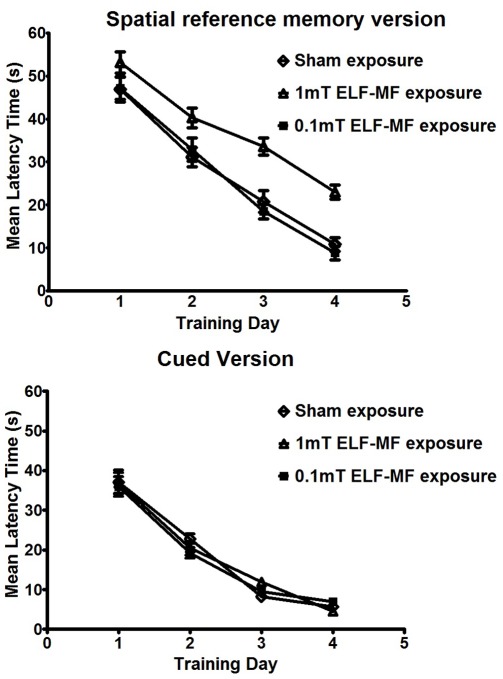
Effect of Electromagnetic Fields Exposure on Two Versions of Water Maze Task. The data represent the latency time to escape to a submersed platform during 4 training days, with four consecutive trials per day. Mice exposed to extremely low frequency magnetic fields (1 mT) took longer to find the platform in the two versions of the water maze compared with the other two groups (p<0.001). No significant deficit in the two versions of the task was observed in the ELF-MF (0.1 mT) exposed mice (p>0.05). Values represent means±SEM. N = 12. * indicates p<0.05.

The effect of ELF-MF exposure on the scores of the mice in the spatial reference memory version of the water maze is presented in Fig. 2. Two-way repeated measures ANOVA indicated a significant effect of group [F(2, 33) = 9.43, P = 0.014], a significant effect of training day [F(3, 99) = 70.6, P<0.001] and a significant interaction between group and training day [F(6, 99) = 20.8, P<0.001]. The effect of ELF-MF exposure on the scores of the mice in the cue memory version of the water maze is presented in Fig. 2. Two-way repeated measures ANOVA indicated a significant effect of group [F(2, 33) = 16.08, P = 0.004], a significant effect of training day [F(3, 99) = 268, P<0.001] and a significant interaction between group and training day [F(6, 99) = 2.923, P = 0.01]. The mean escape latency of the mice improved throughout the training days indicating that the mice were able to learn the two versions of the task. However, the ELF-MF exposure (1 mT) group took longer to find the platform compared with the other two groups (p<0.001). Where as, no significant deficit was observed in the ELF-MF exposure (0.1 mT) mice (p>0.05).

## Discussion

There are many factors that increase ROS concentration in cells, animals and humans. A magnetic field can alter the energy levels and spin orientation of electrons and, as a consequence, increase the activity, concentration and lifetime of free radicals [12], [13], [14]. Oxidative stress results from an imbalance between the generation of free radicals and endogenous protective mechanisms. These protective mechanisms include enzymes that specifically degrade potential ROS precursors, such as CAT that degrades hydrogen-peroxide, and GSH-PX that degrades hydrogen-peroxide in a reaction where reduced glutathione is converted to oxidized glutathione [24].

The present study demonstrated that, in general, exposure to an ELF-MF decreased the activity of antioxidant enzymes and increased the MDA level in two neuronal tissues; the hippocampus and striatum, respectively. The decrease in antioxidant enzyme activity levels might be interpreted as a decrease in protein expression levels in response to the ELF-MF exposure or potentially as the indirect inhibition of the enzymes by their binding with oxidative molecules produced during the ELF-MF exposure. Nonetheless, the GPX and CAT activity decreases may lead to hydrogen-peroxide accumulation, which is favorable to the peroxidation of lipids in tissues [26].

Additionally, the findings of this study show that ELF-MF exposure (50 Hz, 1 mT) impaired the learning of mice in the two versions of the water maze: the spatial reference memory version of the water maze was a declarative memory model which was dependent on the hippocampus [20], [21], and the cue version of the water maze task was a habit learning model which was dependent on the striatum [20], [22]. This study provided direct evidence for the association between the impairment of learning induced by ELF-MF exposure (50 Hz, 1 mT) and oxidative stress in these two brain regions associated with learning and memory.

ROS can activate signal transduction pathways, cause DNA damage, and result in the modification of gene expression. Moreover, the overproduction of oxygen free radicals can give rise to functional and morphological disturbances in the cell through oxidative stress [12], [18], [19]. We speculate that the oxidative stress induced by ELF-MF exposure (50 Hz, 1 mT) may damage the structure and function of hippocampus and striatum in the mice, and therefore impair their ability to generate declarative memory and habit learning.

Yet, the relationship between ELF-MF exposure, oxidative stress and learning impairments is not clearly defined, despite many studies attempting to investigate ELF-MF. For example, Mostafa *et al*. reported that exposure to ELF-MF (50 Hz, 0.2 mT, 24 per day for 1, 2 or 4 weeks) was associated with impairment in discrimination between familiar and novel objects, where as Sienkiewicz *et al*. found no effect of ELF-MF exposure (50 Hz, 75 mT, 45 min, once only) on object discrimination [28], [29]. Lee *et al*. reported that ELF-MF exposure (60 Hz, 1 mT) had no influence on the transformation activities of stress factors such as reactive oxygen species [30]. Our previous results also complicate the issue. We have shown that exposure to ELF-MF (50 Hz,1 mT, 50 min per day) had no effect on response latency of detour learning, but that exposure to ELF-MF (50 Hz, 1 mT, 20 hours per day) significantly delayed detour learning [8]. Nonetheless, the inconsistencies found in the results of these studies may have been due to the differences in the duration of ELF-MF exposure between these studies. Furthermore, the present study showed that the mice of the ELF-MF exposure (1 mT, 4 hours per day) group needed more time to learn the water maze task than the mice of the other two groups and that the development of learning and memory was delayed by ELF-MF (1 mT, 4 hours per day) chronic exposure, where as ELF-MF (0.1 mT, 4 hours per day) chronic exposure did not have an effect on learning or on oxidative stress. All told, the present results, along with the listed previous studies, suggest that the biological effects of ELF-MF exposure depend on the dosage and duration of ELF-MF exposure.

It is also interesting to note that despite the growing body of evidence that links ELF-MF to neurological disfunction and the worrying reports these deleterious effects might have, a small number of studies have suggested beneficial effects of low magnetic or ELF-MF on human health under certain circumstances [31], [32], [33].

Nonethelss, our results do suggest that there may be a negative effect of chronic exposure to ELF-MF (50 Hz, 1 mT, 4 hours per day) on learning and memory, and that this negative effect is associated with oxidative stress. Although, a detailed inquiry into the factors that gave rise to the learning impairment observed here was beyond the scope of the data collected, we believe that the effects of ELF-MF exposure may be complex and subtle and that precise relationship between ELF-MF strength and biological response needs further study.
